# Does neurocognitive function affect cognitive bias toward an emotional stimulus? Association between general attentional ability and attentional bias toward threat

**DOI:** 10.3389/fpsyg.2014.00881

**Published:** 2014-08-12

**Authors:** Yuko Hakamata, Mie Matsui, Hirokuni Tagaya

**Affiliations:** ^1^Department of Clinical Psychology, The University of TokyoTokyo, Japan; ^2^Department of Health Sciences, Kitasato University School of Allied Health SciencesKanagawa, Japan; ^3^Department of Psychology, University of ToyamaToyama, Japan

**Keywords:** attentional bias, neurocognitive function, emotion, attention, cognitive bias

## Abstract

**Background**: Although poorer cognitive performance has been found to be associated with anxiety, it remains unclear whether neurocognitive function affects biased cognitive processing toward emotional information. We investigated whether general cognitive function evaluated with a standard neuropsychological test predicts biased cognition, focusing on attentional bias toward threat.

**Methods**: One hundred and five healthy young adults completed a dot-probe task measuring attentional bias and the Repeatable Battery for the Assessment of Neuropsychological Status (RBANS) measuring general cognitive function, which consists of five domains: immediate memory, visuospatial/constructional, language, attention, and delayed memory. Stepwise multiple regression analysis was performed to examine the relationship between attentional bias and cognitive function.

**Results**: The attentional domain was the best predictor of attentional bias toward threat (β = −0.26, *p* = 0.006). Within the attentional domain, digit symbol coding was negatively correlated with attentional bias (*r* = −0.28, *p* = 0.005).

**Conclusions**: The present study provides the first evidence that general attentional ability, which was assessed with a standard neuropsychological test, affects attentional bias toward threatening information. Individual cognitive profiles might be important for the measurement and modification of cognitive biases.

## Introduction

Cognitive theories suggest that cognitive processing biased toward affective significance confers an increased risk for the development and exacerbation of emotional disorders (Beck, 1976; Mathews and MacLeod, 2005). Such bias is observed in multiple domains of cognition, including greater selective attention toward threats (MacLeod et al., 1986; Fox, 1996), enhanced memory recall of negative stimuli (Gilboa-Schechtman et al., 2002; Ridout et al., 2003), and distorted interpretations of ambiguous information (Lawson et al., 2002; Woud et al., 2014). Indeed, several meta-analytic studies have confirmed the presence of cognitive bias in anxious and depressive individuals (Bar-Haim et al., 2007; Mitte, 2008; Peckham et al., 2010; Phillips et al., 2010), although the exact mechanism for this has yet to be elucidated.

Biased attention toward threatening information is one of the most widely studied cognitive biases. Attentional bias refers to a tendency to quickly identify and easily dwell on emotional stimuli (MacLeod et al., 2002). This process includes visual perception of a stimulus and orientation to it (i.e., selective attention), the sensitivity of which varies among individuals (Posner, 1980; MacLeod et al., 1986). Our previous study found that years of education—a rough estimate of general cognitive function—had an inhibitory effect on attentional bias toward threat, suggesting that one's cognitive ability could affect attentional bias toward threatening stimuli (Hakamata et al., 2013). Despite the dearth of evidence that directly connects general cognitive ability and cognitive biases, several studies have suggested that these two variables might be associated, as they found that some cognitive abilities are generally different in high-anxiety individuals. For example, these individuals had their attention easily diverted by different distractors (Eysenck and Graydon, 1989; Mathews et al., 1990; Eysenck and Byrne, 1992) and showed reduced working memory capacity, even for non-emotional stimuli (Firetto and Davey, 1971; Eysenck, 1979; Darke, 1988; Eysenck et al., 2005; Hayes et al., 2008). Given that cognitive bias is known to be associated with emotional disturbances, such as anxiety (Beck, 1976; Mathews and MacLeod, 2005), these findings raise the possibility that compromised cognitive function facilitates biased cognitive processing toward emotional information. However, no study, to our knowledge, has directly examined the relationship between cognitive bias and cognitive function as assessed with a standard neuropsychological test.

Thus, in the present study, we investigated the relationship between neurocognitive function and cognitive bias, focusing on attentional bias toward threat. We hypothesized that individual attentional function would specifically predict attentional bias toward threat.

## Methods

### Ethical considerations

The Kitasato University Hospital Institutional Review Board approved the study, and all participants provided written informed consent. All the research procedures were conducted in accordance with the Declaration of Helsinki.

### Participants

Participants were 113 individuals recruited via advertisements in a local magazine and billboards at Kitasato University. The eligibility criteria were as follows: no Axis-I psychiatric disorders or substance abuse history, which were determined using the Mini-International Neuropsychiatric Interview (Sheehan et al., 1998), and no major medical/neurological illnesses. Eight subjects were excluded because they had epilepsy (*n* = 2), chronic subdural hematoma (*n* = 1), cerebral palsy (*n* = 1), Wilson disease (*n* = 1), histories of subarachnoid hemorrhage (*n* = 1) and hydrocephalus (*n* = 1), and strabismus (*n* = 1). Thus, data from 105 participants were included in the analyses (63 women, mean age: 22.3 years; range: 20–35, *SD* = 3.2).

### Psychological assessment

#### Anxiety

Anxiety levels were evaluated with the 20 items for trait anxiety from the Spielberger's State-Trait Anxiety Inventory (STAI; Spielberger et al., 1970), a well-established self-report questionnaire measuring anxiety. STAI has been used in previous studies on attentional bias (see Bar-Haim et al., 2007). Each item is rated on a four-point scale (i.e., from 1: “Almost Never” to 4: “Almost Always”), with higher scores indicating greater anxiety. Internal consistency was Cronbach's alpha = 0.86 in the present sample.

#### Depression

Depressive symptoms were evaluated with the Beck Depression Inventory-II (BDI-II). BDI-II is a 21-item, self-report questionnaire to assess depressive symptoms experienced during the past 2 weeks (Beck et al., 1996). Each item is rated on a four-point scale (i.e., from 0 to 3, with higher scores indicating greater severity). Scoring ≤ 17 points on this scale is considered to indicate clinical depression. Internal consistency was Cronbach's alpha = 0.88 in the present sample.

#### Neurocognitive function

The Repeatable Battery for the Assessment of Neuropsychological Status (RBANS) was employed to assess multiple domains of cognitive function. The RBANS is a representative, clinician-administered neuropsychological test for adults aged between 20 and 89 years (Randolph, 1998). It includes 12 standard cognitive subtests, which are grouped into five domains as follows: immediate memory (list learning and story memory), visuospatial/constructional (figure copy and line orientation), language (picture naming and semantic fluency), attention (digit span and digit symbol coding), and delayed memory (list recall, list recognition, story recall, and figure recall). The Japanese version of the RBANS has well-established reliability and validity (Matsui et al., 2010).

To investigate attentional function, the Trail Making Test (TMT) Parts A and B (Reitan, 1992, 1955) were used. Part A requires participants to connect randomly distributed numbers consecutively with a line on paper, and Part B requires participants to connect numbers and letters in an alternating fashion. Response time (RT) indicates visuoperceptual speed and set-shifting ability (i.e., an ability to smoothly switch between different cognitive categories) in Parts A and B, respectively (Strauss et al., 2006).

#### Attentional bias

To measure attentional bias, we used the dot-probe task (DPT), the most commonly used and innovative program for attentional bias modification (MacLeod et al., 1986; MacLeod, 1995). The DPT was constructed on E-prime version 2.0 (Psychology Software Tools, Inc., Pittsburgh, PA). The DPT requires participants to identify a non-emotional probe, such as a letter or symbol (e.g., an asterisk), which can appear in one of two spatial locations. Immediately before probe presentation, threatening and nonthreatening stimuli appear simultaneously in two separate locations. Neutral and negative words were presented as stimuli. We used the word list from the original study by MacLeod et al. (1986). Each trial began with a centrally located fixation cross displayed for 500 ms, followed by a pair of words that appeared vertically on the screen for 500 ms. The words were replaced by an asterisk probe at either the top or bottom location that was just vacated by one of the words. Participants were instructed to press one of two buttons as quickly and accurately as possible to indicate the location of the probe. In total, 196 trials were presented to each participant. The probe replaced the neutral word in half of the trials, appearing on the top and bottom locations of the display with equal probability. The location of the probe was counterbalanced across the experiment. Trial-presentation order was randomized for each participant. Before performing the task, all participants received 32 practice trials on the DPT, using a different set of neutral words. The difference between RT toward neutral stimuli and RT toward negative stimuli serves as an index of attentional bias. Positive values indicate bias toward threat.

### Data analysis

To explore whether a specific cognitive domain affects attentional bias toward threat, we performed a stepwise multiple regression analysis predicting attentional bias, with the five cognitive domains of the RBANS and TMT indices as predictor variables. Effects of age, sex, and years of education were controlled for in the analysis. Inter-correlations were calculated between attentional bias, trait anxiety, depressive symptoms, TMT measures, and the five cognitive domain scores of the RBANS, controlling for age, sex, and years of education. Statistical analyses were performed with SPSS version 22.0J (IBM, Inc., Tokyo, Japan). The significance threshold was set at 0.05 (two-tailed).

## Results

Mean scores and standard deviations (SDs) of the RBANS, TMT, depressive symptoms, trait anxiety, and attentional bias are presented in Table 1. The RBANS and TMT scores in the present study were similar to those of the original studies, except for semantic fluency in the RBANS, the scores of which were relatively lower than those of the original study (Randolph, 1998; Strauss et al., 2006). Inter-correlations between the five cognitive domains of the RBANS, TMT indices, depressive symptoms, trait anxiety, and attentional bias are presented in Table 2. Attentional bias showed significant correlations with the attentional domain of the RBANS and the TMT Part A [*r*_(99)_ = −0.27, *p* = 0.006; *r*_(99)_ = 0.20, *p* = 0.049, respectively], indicating that lower performance in attention-related functions was associated with biased attention toward threatening information. Although attentional bias was not significantly correlated with trait anxiety in the current linear model, its relationship with depressive symptoms bordered on statistical significance (*p* = 0.050).

**Table 1:** **Mean scores and *SD*s of the RBANS subtests, TMT indices, attentional bias, trait anxiety, and depressive symptoms (*N* = 105)**.

	**Mean**	***SD***
Attentional bias	−0.06	13.16
Trait Anxiety (STAI)	43.83	9.10
Depressive symptoms (BDI-II)	7.79	6.72
**RBANS**		
Immediate memory		
List learning	32.10	3.79
Story memory	19.84	3.17
Visuospatial/constructional		
Figure copy	19.45	0.88
Line orientation	18.39	1.72
Language		
Picture naming	9.88	0.36
Semantic fluency	16.89	3.89
Attention		
Digit span	11.90	2.18
Digit symbol coding	63.84	9.01
Delayed memory		
List recall	8.17	1.73
List recognition	19.74	0.59
Story recall	10.98	1.56
Figure recall	17.50	2.58
Total index score	100.52	13.64
**TMT**		
Part A	25.18	7.43
Part B	51.02	12.21

*RBANS, repeatable battery for the assessment of neuropsychological status; STAI, state-trait anxiety inventory; BDI-II, beck depression inventory II; TMT, trail making test*.

**Table 2:** **Correlations between attention bias score, trait anxiety, depressive symptoms, TMT and the RBANS (*N* = 105)**.

				**RBANS**					**TMT**	
	**Attentional bias**	**STAI-T**	**BDI-II**	**Immediate memory**	**Visuospatial/constructional**	**Language**	**Attention**	**Delayed memory**	**Part A**	**Part B**
Attentional bias	–									
Trait anxiety (STAI)	0.09	–								
Depressive symptoms (BDI-II)	0.20^¶^	0.78	–							
**RBANS**										
Immediate memory	−0.01	0.03	0.00	–						
Visuospatial/constructional	0.08	0.02	−0.03	0.09	–					
Language	−0.12	0.00	0.01	0.10	0.10	–				
Attention	−0.27^**^	0.00	−0.05	0.28^**^	0.18	0.10	–			
Delayed memory	0.07	0.01	−0.06	0.64^***^	0.31^***^	0.03	0.19^¶^	–		
**TMT**										
Part A	0.20^*^	0.10	0.11	−0.18	−0.01	−0.12	−0.18	0.03	–	
Part B	0.15	−0.08	−0.06	−0.31^**^	0.04	−0.22^*^	−0.35^***^	−0.16	0.44^***^	–

STAI, state-trait anxiety inventory; BDI-II, beck depression inventory-II;RBANS, repeatable battery for the assessment of neuropsychological status; TMT, trail making test.

¶ *p = 0.05*,

* *p < 0.05*,

** *p < 0.01*,

*** *p < 0.001*.

Next, stepwise multiple regression analysis revealed that the attentional domain of the RBANS was the best predictor of attentional bias toward threat [*F*_(4, 99)_ = 6.06, *R*^2^ = 0.20, adjusted *R*^2^ = 0.16, *p* < 0.001; Table 3]. The model explained 16% of the variance observed. Attentional function negatively affected attentional bias (β = −0.26, *p* = 0.006).

**Table 3:** **Stepwise regression analysis predicting attentional bias toward threat (*N* = 105)**.

**Independent variables**	**Standardized β**	***t***	***p***
**CONTROLLED VARIABLES**			
Age	−0.29	−2.34	0.021
Sex	−0.22	−2.30	0.024
Years of education	−0.08	−0.71	0.481
**SELECTED VARIABLE**			
Attentional domain (RBANS)	−0.26	−2.80	0.006

*Five cognitive domains measured with the Repeatable Battery for the Assessment of Neuropsychological Status (RBANS) (i.e., immediate memory, visuospatial/constructional, language, attention, and delayed memory) and TMT (Trail Making Test) indices (i.e., Parts A and B) were incorporated into the model as predictor variables, controlling for age, sex, and years of education. *R*^2^ = 0.20, adjusted R^2^ = 0.16, p < 0.001. *R*^2^ change = 0.06, p = 0.006 after the attentional domain score of the RBANS was incorporated into the model*.

As a reference analysis to specify which component of the attentional domain in the RBANS was more relevant to attentional bias, partial correlation analysis showed that the digit symbol coding of the attentional domain, not digit span [*r*_(99)_ = −0.12, *p* = 0.236], was negatively correlated with attentional bias toward threat [*r*_(99)_ = −0.28, *p* = 0.005].

Additionally, we performed a confirmatory analysis to examine whether there are gender differences in the RBANS. The ANCOVA, in which age and years of education were controlled for, showed that women had higher immediate memory scores (estimated marginal means: 104.3 ± 1.7 vs. 98.5 ± 2.1, *p* = 0.041) and delayed memory scores (105.7 ± 2.0 vs. 99.2 ± 2.5, *p* = 0.053), compared to men. This is partly consistent with a previous study that found a similar gender difference in the RBANS (Beatty et al., 2003). For the magnitude of the association between the attentional bias and the attentional domain, no significant difference was found between genders (*r* = −0.24 vs. −0.28, *p* = 0.824).

## Discussion

The present study was the first to examine the relationship between cognitive bias and neurocognitive function. The results showed that individuals with lower performance in the attentional domain, particularly in digit symbol coding, exhibited greater attentional bias toward threat, supporting our hypothesis. The significant correlation observed between attentional bias and the TMT Part A further supported the link between attentional function and attentional bias. These findings suggest that general attentional ability could affect biased attention toward threatening information.

It is important to note that the digit symbol coding test, which has been widely used to assess attentional resources as a part of working memory (Goldman-Rakic, 1994; Stratta et al., 1997; Pukrop et al., 2003), was significantly associated with attentional bias. Digit symbol performance is considered to be influenced by different cognitive components (Lezak et al., 2004), although recent evidence suggests that its primary component is visuoperceptual speed (Joy et al., 2004). Digit symbol coding—but not digit span, which specifically requires auditory inputs—was correlated with attentional bias. This suggests that the visuoperceptual facet of attention, some variance of which also overlaps with visuospatial working memory, might be more relevant to biased attention. This is in line with the significant correlation found between attentional bias and visuoperceptual speed as measured by the TMT Part A.

Recent neuroimaging research indicates that attentional bias toward threat is associated with activity in the dorsolateral prefrontal cortex (DLPFC) (Fani et al., 2012; Peers et al., 2013; Brunoni and Vanderhasselt, 2014). In accordance with this evidence, an fMRI study examining neutral activity during the digit symbol test observed DLPFC activation (Usui et al., 2009). The DLPFC has been implicated in the allocation of visuospatial attention (Makino et al., 2004; Anderson et al., 2007) as well as in working memory function (Wager and Smith, 2003; Brunoni and Vanderhasselt, 2014). These findings support the idea that attentional bias might share neural underpinnings with visuoperceptual facets of attention, and that the two systems functionally interact with one another. Compromised attentional ability, particularly of visuoperceptual facets, might affect biased selective visual attention toward negative information.

Some limitations should be noted when interpreting the results. First, we used the RBANS to measure neurocognitive function. More detailed assessment tools for assessing neurocognitive function are necessary. Second, the semantic fluency scores in the present study were lower than the scores in the original study (Randolph, 1998; Strauss et al., 2006). Although this difference did not affect the main results, this point should be considered as a limitation. Third, a significant association between anxiety and attentional bias was not observed, whereas the association between depressive symptoms and attentional bias was partially demonstrated, as it was in previous meta-analytic studies (Peckham et al., 2010; Phillips et al., 2010). This might have been caused by insufficient statistical power to detect this association, given that the reported effect sizes for the relation between these two variables are not large and that they have been calculated from two-group comparisons between high-anxiety and low-anxiety participants, usually defined as scoring 1 *SD* above and below the mean on anxiety-related measures, respectively. In future studies, a prospective design using a larger sample is needed to determine whether poorer attentional function precedes greater attentional bias, while considering a variety of other potential intervening variables (e.g., gender).

In summary, we revealed that general attentional ability, assessed with a standard neuropsychological test, affects attentional bias toward threat. Compromised neurocognitive function in a specific domain might affect biased cognition toward an emotional stimulus therein. Consideration of individual differences in neurocognitive function might be important for the measurement and modification of cognitive bias.

### Conflict of interest statement

The authors declare that the research was conducted in the absence of any commercial or financial relationships that could be construed as a potential conflict of interest.
